# Assessment of Paraquat Resistance and Degradation Potential in *Caballeronia zhejiangensis* CEIB S4-3: The Genomic Analysis Reveals Hints About Resistance and Degradation Mechanisms

**DOI:** 10.3390/toxics14050405

**Published:** 2026-05-08

**Authors:** Manuel Isaac Morales-Olivares, María Luisa Castrejón-Godínez, Patricia Mussali-Galante, Efraín Tovar-Sánchez, Alexis Rodríguez

**Affiliations:** 1 Programa de Doctorado en Ciencias Naturales, Universidad Autónoma del Estado de Morelos, Av. Universidad 1001, Col. Chamilpa, Cuernavaca C.P. 62209, Morelos, Mexico; manuel.morales@fcbiologicas.uaem.edu.mx; 2 Facultad de Ciencias Biológicas, Universidad Autónoma del Estado de Morelos, Av. Universidad 1001, Col. Chamilpa, Cuernavaca C.P. 62209, Morelos, Mexico; 3 Centro de Investigación en Biotecnología, Universidad Autónoma del Estado de Morelos, Av. Universidad 1001, Col. Chamilpa, Cuernavaca C.P. 62209, Morelos, Mexico; patricia.mussali@uaem.mx; 4 Centro de Investigación en Biodiversidad y Conservación, Universidad Autónoma del Estado de Morelos, Av. Universidad 1001, Col. Chamilpa, Cuernavaca C.P. 62209, Morelos, Mexico; efrain_tovar@uaem.mx

**Keywords:** bacterial resistance, biodegradation, bioremediation, environmental pollution, herbicide, pesticides

## Abstract

Paraquat is an herbicide widely used to control weeds in various crops. Due to its use in large quantities, its dispersal into the environment is frequent, leading to contamination and negative health effects on non-target organisms because of its high toxicity and persistence in soils. Therefore, it is necessary to develop sustainable strategies to remediate sites contaminated by this compound. Bacterial remediation is a promising alternative for removing paraquat from the environment; however, the metabolic pathways used by bacteria for its degradation have not yet been precisely described. In this context, it is essential to characterize bacterial species capable of resisting and degrading paraquat, as well as to elucidate the molecular mechanisms involved in these processes. The objective of this work was to evaluate the paraquat resistance and degradation potential of the bacterial strain *Caballeronia zhejiangensis* CEIB S4-3, and to identify genes with a possible role in the resistance and degradation of this herbicide by analyzing the strain’s genome. The results of this research showed that, in solid medium, *C. zhejiangensis* CEIB S4-3 can withstand concentrations of up to 200 mg/L of paraquat supplemented as a commercial formulation (Gramoxone^®^) and 400 mg/L of analytical-grade paraquat. In tryptic soy broth, the strain grew in the presence of both the commercial formulation and analytical-grade paraquat at concentrations up to 15 mg/L, whereas in mineral salts medium, supplemented with paraquat or its commercial formulation as the sole nutrient source, the strain survived exposure to paraquat at the same concentrations. Furthermore, the bacterial strain removed 40.8% of the paraquat supplemented in the culture medium at a concentration of 12 mg/L within 48 h. Finally, genomic analysis revealed the presence of genes related to paraquat resistance mechanisms and encoding enzymes involved in the degradation of this herbicide. These results position *C. zhejiangensis* CEIB S4-3 as a promising candidate for developing remediation alternatives for sites contaminated with this herbicide.

## 1. Introduction

Agriculture is a primary economic activity essential for meeting the food needs of a constantly growing population [1]. To maintain productivity, modern agricultural systems rely on a wide variety of pesticides that help reduce losses caused by pests [2]. Recent data from the Food and Agriculture Organization (FAO) of the United Nations indicate that, in 2023 alone, 3,728,427 tons of pesticides were used globally, of which 2,009,099 (53.89%) tons were herbicides. That same year, approximately 8859 tons of bipyridyl herbicides were used, including paraquat [3,4], one of the most widely used herbicides in the agricultural sector.

Paraquat (1,1′-dimethyl-4,4′-bipyridyl dichloride) is a non-selective contact herbicide widely used for its effectiveness, low cost, and high commercial availability [5,6,7], which has made it the third most used herbicide worldwide, especially in developing countries [8,9]. Paraquat is used for weed control in peanut, coffee, corn, oil palm, and banana crops [10,11,12,13,14], as well as in defoliation in cotton, sugarcane, bean, potato, and soybean crops [15,16,17,18,19].

After its agricultural application, paraquat can disperse into the environment and contaminate soil, surface, and groundwater [20]. Due to this, paraquat is frequently identified in agricultural and surrounding areas in many regions; concentrations of up to 15 mg/kg have been documented in agricultural soils in Mérida, Venezuela [21], 134 µg/L in surface waters in Mai Chau Province, Vietnam [22], and 22 µg/L in groundwater in Thailand [23]. Its high toxicity contrasts with that of other herbicides; while the median lethal dose (LD_50_) of glyphosate and atrazine in *Rattus norvegicus* is 7444 mg/kg and 3090 mg/kg, respectively [24,25], that of paraquat is only 125 mg/kg [26].

The presence of paraquat in soils affects microbial communities, reducing populations responsible for essential processes such as organic matter decomposition and nutrient cycling [27,28,29]. Likewise, in aquatic environments, the presence of paraquat compromises the survival of invertebrates such as springtails and shrimps [30,31,32], in addition to generating teratogenic effects in fish and amphibians [33,34,35,36]. Liver and kidney damage, as well as genetic damage such as DNA breaks, have been documented in Swiss albino mice [37], while in humans, acute paraquat intoxication may cause lung injury and, in severe cases, death [38], but chronic exposure could be related to the development of neurodegenerative diseases and cancer [38,39].

In response to this problem, physicochemical methods have been implemented for paraquat removal, such as adsorption using biochar or montmorillonite, which have negatively charged carboxyl and hydroxyl functional groups that effectively retain cationic compounds like paraquat [40,41]. Cellulose triacetate membranes have also been developed to separate the herbicide from aqueous solutions [42], as well as advanced oxidation processes, such as photo-Fenton, capable of completely mineralizing it [43]. However, these techniques often require expensive equipment, are difficult to scale up, can generate secondary hazardous waste, and do not consistently achieve complete removal or degradation [44].

Bacterial bioremediation has gained relevance due to its ability to leverage enzymatic machinery to transform or eliminate herbicides that pollute the environment [45]. This approach not only reduces reliance on expensive or difficult-to-implement technologies but also offers a sustainable alternative, compatible with natural ecosystem processes, for eliminating pollutants. Several bacterial strains capable of degrading paraquat have been reported, with species from the genera *Bacillus* and *Pseudomonas* among the most studied [46]. However, the efficiency of bioremediation largely depends on understanding the cellular and metabolic mechanisms that allow various bacteria to tolerate, transform, or degrade highly toxic compounds such as paraquat.

Paraquat is a highly toxic molecule that causes cellular death due to the induction of exacerbated oxidative stress mediated by reactive oxygen species such as superoxide (O_2_^−^) and hydroperoxide (H_2_O_2_) radicals [47]. Bacteria exposed to paraquat can counteract its toxicity through different resistance mechanisms such as (1) oxidative stress sensing and subsequent overexpression of antioxidant enzymes (i.e., superoxide dismutases, catalases, flavodoxins and peroxidases) mediated by regulatory systems such as the regulons SoxRS and OxyRS, (2) the paraquat expulsion out of the cell through efflux systems (i.e., multidrug efflux pumps from different families such as MFS or RND) or (3) biodegradation, mediated by oxidative enzymes such as cytochrome P450, monooxygenases and dioxygenases [46,48,49,50].

Despite advances, the mechanisms of paraquat biodegradation remain poorly understood, limiting the application of microorganisms’ biotechnological potential to counteract paraquat pollution. Therefore, analyzing genomic information from pesticide-degrading bacterial strains may help better understand the biochemical processes involved in the removal of pesticides such as paraquat [51].

These knowledge gaps highlight the need for further research to elucidate the cellular and biochemical processes through which bacteria can remove or degrade paraquat. In this regard, the use of bacteria previously reported for their broad capacity to degrade other pesticides, such as *Caballeronia zhejiangensis* CEIB S4-3, is a promising alternative for evaluating their paraquat removal and degradation capacity. This strain has been shown to hydrolyze the organophosphate pesticide methyl parathion (50 mg/L) and completely degrade its main hydrolysis product, *p*-nitrophenol, within 12 h [52,53]. The strain is also capable of degrading 60% of the herbicide glyphosate at the same concentration and timeframe [54]. In this context, the present study aimed to evaluate the ability of *C. zhejiangensis* CEIB S4-3 to resist and degrade paraquat and to analyze its genome to provide scientific knowledge that contributes to elucidating the biochemical and cellular processes involved in its bioremediation.

## 2. Materials and Methods

### 2.1. Paraquat

Two sources of paraquat were used: a commercial formulation containing 200 g/L paraquat (Gramoxone^®^, Syngenta Agro S.A. de C.V., San Luis Potosí, S.L.P., Mexico) and analytical-grade paraquat with 99.5% purity (Chem Service Incorporated, West Chester, PA, USA). To prepare the paraquat stock solutions (50,000 mg/L), both the commercial formulation and analytical-grade reagent were diluted in sterile Milli-Q water. Stock solutions were used to prepare the solutions required for subsequent experiments.

### 2.2. Bacterial Strain and Culture Media

The *Caballeronia zhejiangensis* CEIB S4-3 strain was cultured on Petri dishes containing tryptic soy agar (TSA) and incubated at 30 °C for 72 h. Subsequently, isolated colonies were transferred to 250 mL Erlenmeyer flasks containing 50 mL of tryptic soy broth (TSB) (Bioxon, Becton Dickinson de México S.A. de C.V., Cuautitlán Izcalli, State of Mexico, Mexico) and incubated at 30 °C for 24 h with shaking at 150 rpm.

The bacterial cultures in TSB were transferred to 50 mL conical tubes and centrifuged for 10 min at 3500 rpm. Subsequently, the supernatants were decanted and the resulting bacterial biomass was washed with 5 mL of Mineral Salts Medium (MSM), whose composition per liter consists of 998 mL of solution A (KH_2_PO_4_ 0.82 g; K_2_HPO_4_ 0.19 g; MgSO_4_ 7H_2_O 0.20 g; KNO_3_ 2 g and (NH_4_)_2_SO_4_ 0.99 g) and 2 mL of solution B (H_3_BO_3_ 2.8 g; MnSO_4_ H_2_O 2.55 g; CuSO_4_ 5H_2_O 0.17 g; CoCl_2_ 6H_2_O 2.43 g and ZnSO_4_ 7H_2_O 0.25 g). The washing and centrifugation processes were carried out twice, and, depending on the experiment, the inoculum was prepared by resuspending the bacterial biomass in sterile TSB or MSM [54].

### 2.3. Determination of Paraquat Bacterial Resistance: Minimum Inhibitory Concentration on Agar Plates

To evaluate the resistance of *C. zhejiangensis* CEIB S4-3 to Gramoxone^®^ or analytical-grade paraquat on agar plates, sterile conical tubes were used to mix 1 mL of the bacterial inoculum in TSB, adjusted to an optical density (OD_600 nm_) of 0.5, with 9 mL of warm TSA. The mixture was manually homogenized to favor cell distribution and poured into Petri dishes. Subsequently, sterile filter paper discs (5 mm in diameter) were placed on the surface of the solidified plates, and 5 μL of Gramoxone^®^ or analytical-grade paraquat solutions at concentrations of 25, 50, 100, 200, 400, 800, 1600, 3200, 6400, and 12,800 mg/L were added to each disc. Sterile Milli-Q water was used as a negative control of bacterial growth inhibition, while the commercial formulation Gramoxone^®^ (200 g/L) without dilution was used as a positive inhibition control. Petri dishes were incubated at 30 °C for 48 h, and the diameters of the inhibition halos were measured at 24 and 48 h. Three biological replicates were performed in each experiment [54].

### 2.4. Determination of Paraquat Bacterial Resistance and Growth Inhibition in Tryptic Soy Broth

To evaluate the effect of exposure to Gramoxone^®^ or analytical-grade paraquat on the growth of *C. zhejiangensis* CEIB S4-3 in liquid cultures, 250 mL Erlenmeyer flasks containing 50 mL of TSB were inoculated with the bacterial strain at an OD_600 nm_ of 0.05. Subsequently, the culture medium was supplemented with Gramoxone^®^ or analytical-grade paraquat at concentrations of 1, 3, 5, 10, and 15 mg/L. Bacterial cultures without paraquat were used as growth control, while TSB was used as a blank. All cultures were incubated at 30 °C and shaken at 150 rpm for 72 h. Bacterial growth was determined through OD_600 nm_ measurements at 0, 4, 8, 12, 24, 36, 48, 60, and 72 h. Each experiment was carried out with three biological replicates [54].

### 2.5. Effect of Paraquat Exposure on Minimal Salts Medium

To evaluate the effect of exposure to Gramoxone^®^ or analytical-grade paraquat on the cell density of *C. zhejiangensis* CEIB S4-3 in MSM, 250 mL Erlenmeyer flasks containing 50 mL of MSM supplemented with 1, 3, 5, 10, and 15 mg/L of Gramoxone^®^ or analytical-grade paraquat were used. The flasks were inoculated with the bacterial strain at an OD_600 nm_ of 0.5 and incubated at 30 °C and 150 rpm. Samples were collected at 0, 4, 8, 12, 24, 36, 48, 60, and 72 h, and the OD_600 nm_ of the cultures was measured. Each experiment was performed in triplicate [54].

### 2.6. Paraquat Removal Kinetics

Paraquat removal assays were carried out in 125 mL containers supplemented with 12 mg/L of paraquat and a bacterial inoculum adjusted to an OD_600 nm_ of 1. The samples were incubated under agitation (150 rpm) for 72 h at 30 °C. To determine the removal of the paraquat herbicide, 2 mL samples were collected every 24 h until the end of the kinetics. The samples were centrifuged at 4000 rpm for 10 min, and then the supernatants were separated from the cellular precipitate by decantation and filtered using a nylon syringe filter with a diameter of 13 mm and pores of 0.2 µm. A control, MSM supplemented with paraquat (12 mg/L), was included in the experiment to rule out potential degradation due to abiotic factors.

For paraquat measurement, the filtered samples were injected into an Ultra High Performance Liquid Chromatography equipment equipped with a diode array detector (UHPLC, Dionex UltiMate 3000, Thermo Scientific, Sunnyvale, CA, USA), using an Agilent InfinityLab Poroshell 120 EC-C18 column (2.1 mm × 30 mm, 2.7 µm; Agilent Technologies, Inc., Santa Clara, CA, USA), the 5 min chromatographic runs were isocratic with the following mobile phase: phosphate buffer (0.05 M) + acetonitrile (65:35% v.v.), at a flow rate of 1 mL/min and detection at a wavelength of 257 nm. Finally, the residual paraquat concentration was determined using a calibration curve generated with analytical-grade paraquat at concentrations ranging from 0 to 25 mg/L (Figure S1).

### 2.7. Genomic Analyses

Different bioinformatics tools were employed to analyze the draft genome of *C. zhejiangensis* CEIB S4-3 strain, which is publicly available in the NCBI-GenBank database (https://www.ncbi.nlm.nih.gov/ (accessed on 18 March 2026)) under the accession number JSBM00000000 and the BioProject: PRJNA264584, and to identify genes involved in the paraquat resistance and biodegradation mechanisms in the *C. zhejiangensis* CEIB S4-3 genome. Genomic sequence search was conducted using sequences of orthologous genes and proteins from species closely related to the *C. zhejiangensis* CEIB S4-3 bacterial strain (*Burkholderia*, *Caballeronia*, and *Pseudomonas*), reported in paraquat resistance and degradation mechanisms. Gene identification was performed by translating the protein sequences into their corresponding nucleotide sequences, followed by sequence alignments using the TBlastn tool in the Basic Local Alignment Search Tool (BLAST, https://blast.ncbi.nlm.nih.gov/Blast.cgi (accessed on 18 March 2026)). The Clusters of Orthologous Genes (COGs) were identified through BLASTX analysis using the nucleotide sequences of the genes and the reference the closed genome of the strain *Caballeronia zhejiangensis* A33_M4_a (NCBI BioProject: PRJNA765207) in the JGI genome portal, Integrated Microbial Genomes and Microbiomes (JGI IMG/M; https://img.jgi.doe.gov/ (accessed on 22 April 2026)), the identified COG of each gene was subsequently used for functional identification (KO) in the Kyoto Encyclopedia of Genes and Genomes (KEGG; https://www.genome.jp/kegg/ (accessed on 23 April 2026)).

### 2.8. Statistical Analyses

The dataset obtained from the experimental results was processed using a Shapiro–Wilk test to assess normality and a Brown–Forsythe test to assess homogeneity of variance in the STATISTICA version 8.0 software [55]. Subsequently, once the normality of the data and the homogeneity of variance were determined—Shapiro–Wilk (*p* > 0.05) and Brown–Forsythe (*F*_53, 108_ = 06043; *p* = 0.8112)—a two-way analysis of variance (ANOVA) was performed to assess the effect of paraquat at different concentrations on the growth of the bacterial strain (OD_600_ nm). At the same time, a post hoc Tukey test (*p* < 0.05) was conducted to determine significant differences in the growth (OD_600 nm_) of the bacterial strain over time in response to paraquat exposure. Significant differences in paraquat concentration over time in the removal kinetics were determined using one-way ANOVA followed by post hoc Tukey test (*p* < 0.05). Variance analyses (ANOVA) were conducted using Prism GraphPad V 8.0.2 statistical tool (GraphPad Software Inc.; San Diego, CA, USA).

## 3. Results

### 3.1. Gramoxone^®^ and Analytical-Grade Paraquat Minimum Inhibitory Concentration Assays on Tryptic Soy Agar Plates

**Figure 1:**
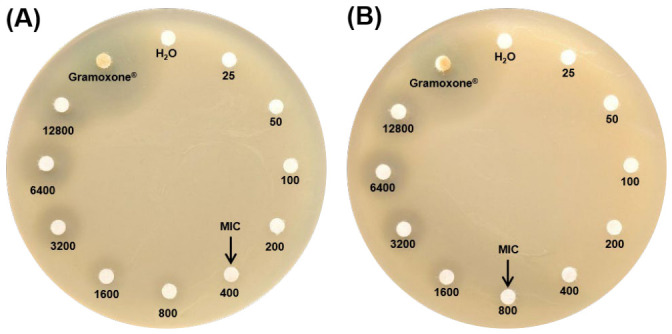
Paraquat minimum inhibitory concentration (MIC) on agar plates. (**A**) Paraquat based commercial herbicide formulation (Gramoxone^®^), and (**B**) analytical-grade paraquat. Milli-Q water was used as the negative control for bacterial growth inhibition, and the Gramoxone^®^ formulation (200 g/L) without dilution was used as the positive control for bacterial growth inhibition. The concentration range used to determine the MIC was 25–12,800 mg/L. The black arrows indicate the Minimum Inhibitory Concentration (MIC).

To evaluate the resistance of the strain *C. zhejiangensis* CEIB S4-3 to paraquat, bacterial cultures in tryptic soy agar plates were exposed to increasing concentrations (25–12, 800 mg/L) of paraquat, using the commercial herbicide formulation (Gramoxone^®^) (Figure 1A) and the analytical-grade (98% purity) paraquat molecule (Figure 1B). As shown in panel A of Figure 1, the bacterial strain *C. zhejiangensis* CEIB S4-3 was resistant to paraquat present in the commercial herbicide formulation at concentrations of 25–200 mg/L, whereas exposure at 400–12,800 mg/L resulted in the formation of bacterial growth-inhibition halos on the agar plates. The bacterial strain was resistant to paraquat (analytical-grade) at concentrations of 25–800 mg/L, whereas concentrations above caused the development of bacterial inhibition halos on the agar plates (Figure 1B). According to these findings, *C. zhejiangensis* CEIB S4-3 growing in solid culture was able to tolerate high concentrations of paraquat in both commercial herbicide formulations and the pure compound.

### 3.2. Gramoxone^®^ and Analytical-Grade Paraquat Bacterial Growth Inhibition Assays in Tryptic Soy Broth

To determine the toxic effects of paraquat exposure on the bacterial growth of the strain *C. zhejiangensis* CEIB S4-3 in liquid media, cultures supplemented with concentrations of 1, 3, 5, 10, and 15 mg/L were prepared using the commercial herbicide formulation Gramoxone^®^ and the analytical-grade paraquat (98% purity).

**Figure 2:**
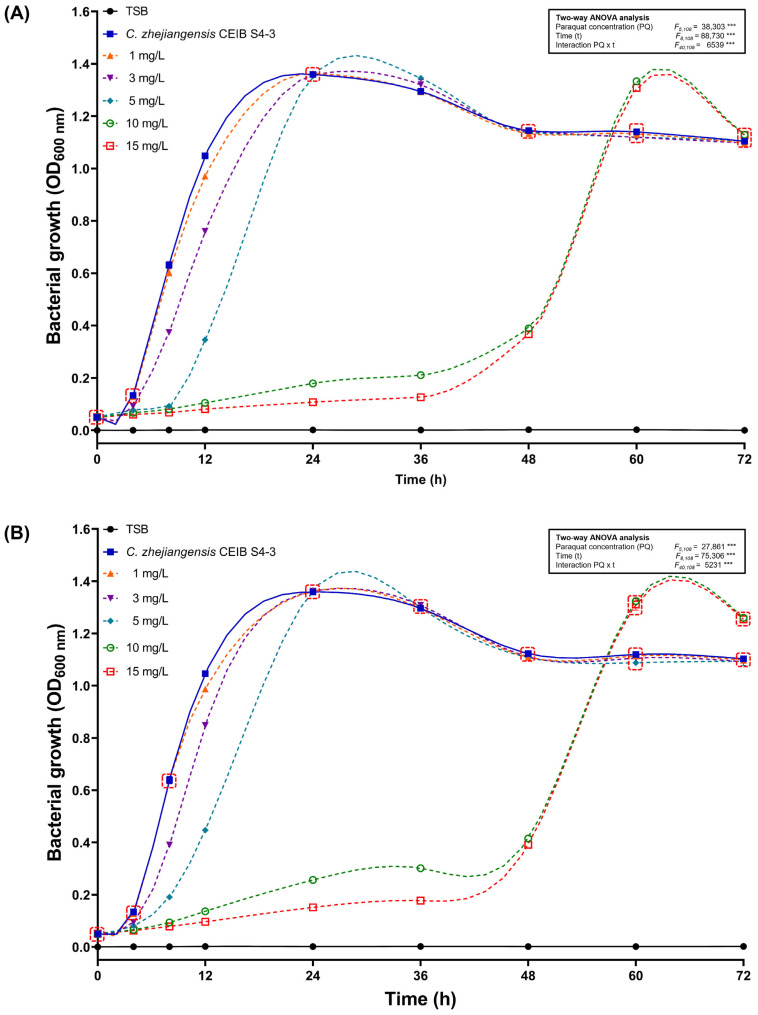
Growth kinetics of *C. zhejiangensis* CEIB S4-3 in tryptic soy broth supplemented with (**A**) Gramoxone^®^ and (**B**) Analytical-grade paraquat. Three replicates were performed in all experiments. Markers represent average values, while error bars show standard deviation. Two-way ANOVA test: *** *p* ≤ 0.001. Points within the dotted red boxes did not show statistically significant differences among themselves (Tukey post hoc test, *p* > 0.05).

According to the results of the bacterial growth inhibition assays, exposure to the commercial herbicide formulation Gramoxone^®^ resulted in adverse effects on the growth of the strain *C. zhejiangensis* CEIB S4-3 compared to cultures without herbicide. As shown in Figure 2A, exposure to herbicide formulation generated bacteriostatic effects on cell growth; the observed inhibitory effects were dependent on the concentration of the paraquat present in the cultures. Exposure to 1, 3, and 5 mg/L of paraquat showed inhibitory effects only during the first 24 h of incubation; subsequently, no statistically significant differences in OD_600 nm_ were observed compared with the cultures without herbicide. On the other hand, exposure to concentrations of 10 and 15 mg/L of paraquat caused the greatest bacterial growth inhibitory effects, as result, the strain entered the exponential growth phase after 48 h of incubation and reached its maximum OD_600 nm_ around 60 h, while cultures in absence of herbicide quickly started the exponential growth phase and reached the maximum OD_600 nm_ at the 24 h of incubation.

Similar findings were observed in the bacterial cultures supplemented with paraquat (analytical-grade). Exposure to the lower paraquat concentrations evaluated (1–5 mg/L) produced a slight bacteriostatic effect only during the first 24 h of incubation; thereafter, the observed OD_600 nm_ values did not show statistical differences compared to the values measured for cultures in the absence of paraquat. In higher paraquat concentrations (10 and 15 mg/L) a strong bacteriostatic effect was observed during the first 36 h of the growth kinetics, subsequently cultures increased gradually their OD_600 nm_ in the 36–48 h incubation lapse, and then the exponential growth phase was observed from 48 to 60 h, the highest OD_600 nm_ was measured around 60 h (Figure 2B). These results indicate that, despite the high toxicity of paraquat to the *C. zhejiangensis* CEIB S4-3 strain, especially at concentrations of 10 and 15 mg/L, the strain was not completely inhibited in its growth and was able to resist the exposure to this herbicide.

### 3.3. Exposure Kinetics to Gramoxone^®^ and Analytical-Grade Paraquat in Mineral Salt Medium

To determine the effect of paraquat exposure on bacterial cultures of *C. zhejiangensis* CEIB S4-3 (0.5 OD_600 nm_) in mineral salt media (MSM) without the presence of additional carbon sources, exposure kinetics were performed for 72 h supplementing concentrations of 1, 3, 5, 10 and 15 mg/L of paraquat from the commercial formulation of the herbicide (Gramoxone^®^) and as an analytical-grade molecule.

**Figure 3:**
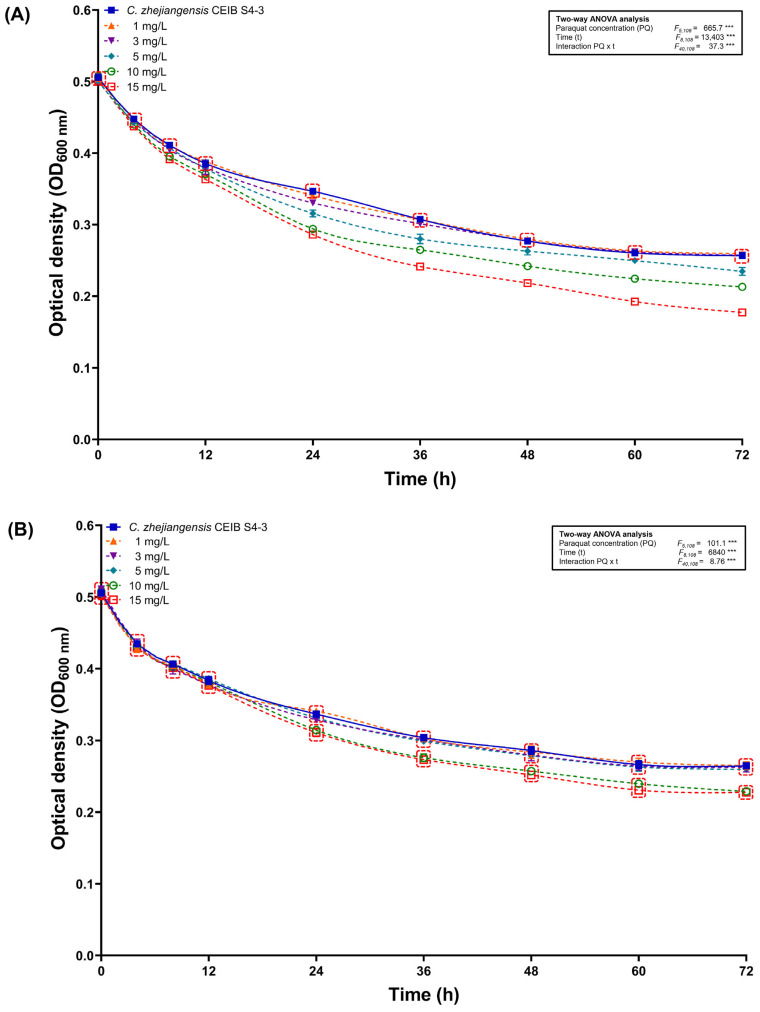
Exposure kinetics of *C. zhejiangensis* CEIB S4-3 in minimal salts medium supplemented with (**A**) Gramoxone^®^ and (**B**) analytical-grade paraquat. Three replicates were performed in all experiments. Markers represent average values, while error bars show standard deviation. Two-way ANOVA test: *** *p* ≤ 0.001. Points within the red boxes did not show statistically significant differences among themselves (Tukey post hoc test, *p* > 0.05).

During the paraquat exposure kinetics using Gramoxone^®^ (Figure 3A), it was observed that all bacterial cultures could survive in the presence of the different paraquat concentrations evaluated. During the first 12 h of exposure to concentrations of 1, 3, and 5 mg/L, the OD_600 nm_ of the cultures did not differ significantly from the values measured for bacterial cultures in the absence of paraquat. However, at higher concentrations of 10 and 15 mg/L, a significant decrease in OD_600 nm_ (4.6 and 5.7%, respectively) was observed compared to the OD_600 nm_ values measured for cultures without herbicide. Finally, after 72 h of exposure, the OD_600 nm_ of the cultures in the presence of paraquat (Gramoxone^®^) at concentrations of 1 and 3 mg/L did not differ significantly from that observed for cultures without herbicide. On the other hand, in the bacterial cultures exposed to concentrations of 5, 10 and 15 mg/L of paraquat (Gramoxone^®^), decreases in OD_600 nm_ of 8.6, 17.97 and 30.9% were observed, respectively.

On the other hand, in the exposure kinetics to analytical grade paraquat (Figure 3B), similar results to those of the exposure kinetics to the commercial herbicide formulation were observed. During the first 12 h of kinetics, the OD_600 nm_ of all bacterial cultures exposed to paraquat showed no significant differences compared to the control without herbicide. Subsequently, after 72 h of incubation, the OD_600 nm_ of the cultures exposed to concentrations of 1, 3, and 5 mg/L of paraquat (analytical grade) did not differ statistically from the values measured in the control cultures (without paraquat), while at concentrations of 10 and 15 mg/L, the OD_600 nm_ decreased by 13.6 and 13.98%, respectively, compared to the cultures without the presence of paraquat. Finally, the bacterial cultures were able to withstand all paraquat concentrations evaluated in MSM without additional carbon sources.

### 3.4. Paraquat Removal Kinetics

**Figure 4:**
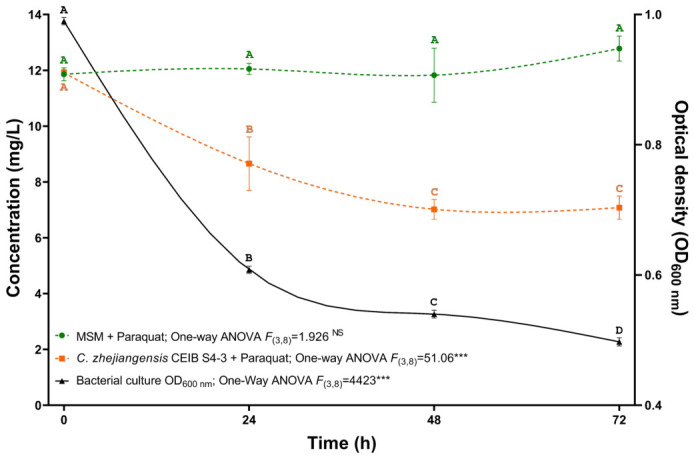
Paraquat removal kinetics. Bacterial strain *C. zhejiangensis* CEIB S4-3 was inoculated at an initial OD_600 nm_ of 1.0. Three replicates were performed in all experiments. Markers represent average values, while error bars show standard deviation. One-way ANOVA test: ^NS^ *p* > 0.05; *** *p* ≤ 0.001. In each treatment, different letters indicate statistically significant differences (Tukey post hoc test, *p* ≤ 0.05).

To determine the capacity of the *C. zhejiangensis* CEIB S4-3 strain to remove paraquat in vitro, removal kinetics were performed in MSM supplemented with 12 mg/L of analytical-grade paraquat (UHPLC chromatograms are shown in Figures S2 and S3). According to the results of these experiments, the *C. zhejiangensis* CEIB S4-3 strain reduced the herbicide concentration. As shown in Figure 4, the strain exhibited an initial paraquat removal of 27% within the first 24 h. Subsequently, the paraquat concentration in the medium decreased by 40.83% after 48 h. At the following monitoring time, 72 h, the paraquat concentration did not decrease significantly compared to the removal levels determined at 48 h. This could be associated with: 1. the depletion of readily bioavailable paraquat or the accumulation of transformation intermediates that could inhibit further degradation; 2. oxidative stress caused by paraquat exposure, leading to reduced microbial activity despite the presence of stress response genes; and 3. metabolic or energetic limitations, where key degradation pathways are downregulated once bacterial cells enter the stationary phase. Furthermore, genomic data suggest that while genes related to redox balance and stress tolerance exist, there is limited evidence of a complete and highly efficient paraquat mineralization pathway, which could limit sustained degradation over time.

As shown in Figure 4, the removal kinetics began with a bacterial inoculum at an OD_600 nm_ of 1.0. However, after the first 24 h of incubation, the OD_600 nm_ decreased significantly (38.6%) compared to the time 0 h. At the 48 and 72 h monitoring times, the OD_600 nm_ of the culture decreased by 45.45 and 49.76%, respectively. This reduction in bacterial density can be attributed to both paraquat toxicity and MMS use. Paraquat induces oxidative stress by generating reactive oxygen species, which can damage cellular components. Furthermore, the metabolic burden associated with the stress response and detoxification mechanisms can further reduce bacterial density. Additionally, the absence of readily available carbon and energy sources in MSM may limit bacterial growth and metabolic activity, forcing cells to rely on paraquat as their sole nutrient source. The decreases in the OD_600 nm_ of the bacterial cultures observed during the development of the removal kinetics could have affected the paraquat herbicide removal process in the system.

### 3.5. Identification of Genes Implicated in the Resistance and Degradation of Paraquat Through the C. yhejiangensis CEIB S4-3 Genome Analysis

The analysis of the genome of the *C. zhejiangensis* CEIB S4-3 strain allowed the identification of different genetic sequences related to paraquat resistance and degradation potential (Table 1). The identified genetic sequences can be divided into four general molecular strategies to deal with adverse effects derived from exposure to paraquat: (1) containment of oxidative stress, (2) efflux systems, (3) remodeling and repair of the bacterial membrane, and (4) degradation of the herbicide.

Regarding sequences related to the containment of oxidative stress generated by exposure to paraquat, sequences of transcriptional regulators such as *soxR*, *soxS*, *oxyR* and *oxyS* were identified, reported as important for sensing oxidative stress related to the production of reactive oxygen species such as superoxide (O_2_^−^) and peroxide radicals (H_2_O_2_) generated by exposure to paraquat, as well as with the activation of the oxidative stress response in bacteria, through the induction of the expression of antioxidant enzymes, efflux systems and the repair of oxidative damage. Sequences that encode antioxidant enzymes were also identified in the analysis of the genome, among them, superoxide dismutases, catalases, flavodoxins and peroxiredoxins.

Efflux systems play an important role in the detoxification of different toxic substances, such as antibiotics and other xenobiotics, including pesticides. In the *C. zhejiangensis* CEIB S4-3 genome, different sequences encoding transporters from the Major Facilitator Superfamily (MFS) and the Resistance–Nodulation–Division (RND) transporter family were identified. On the other hand, paraquat exposure can cause oxidative damage to the inner membrane in bacteria. In this study, three genes that encode for the transmembrane protein system formed by the paraquat inducible proteins A, B and C (*pqi*A, *pqi*B, and *pqi*C) were identified. This protein system (PqiABC) is involved in the transport of lipids from the external to the internal cell membrane, which allows the remodeling and repair of the bacterial membrane in response to oxidative stress and lipid peroxidation induced by exposure to paraquat, thereby reducing damage to the bacterial membrane.

Finally, several genes that encode enzymes reported with potential for the degradation of paraquat herbicides were identified. Among them, the cytochrome P450 enzymes, reported in the N-demethylation of paraquat, flavin-monoxygenases implicated in processes such as N-demethylation, hydroxylation or the initial oxidative modification of the bipyridyl structure, before ring cleavage reactions, as well as dioxygenases, enzymes that could open the aromatic ring and cleave the paraquat molecule into smaller organic acids. Furthermore, the sequence of a Dyp-peroxidase was identified in the genome. This heme-dependent peroxidase can degrade complex aromatic structures, including those found in synthetic colorants, lignin, and aromatic contaminants.

## 4. Discussion

The herbicide paraquat is a bipyridyl compound widely used in agriculture, whose persistence and high toxicity have raised significant concerns regarding its environmental impact. Consequently, there has been increasing interest in bioremediation strategies based on microorganisms capable of resisting and degrading this compound [29,49]. Several studies have demonstrated that certain bacteria can develop resistance to paraquat and are able to transform or degrade the herbicide, particularly those isolated from agricultural soils with a long history of pesticide exposure [91,92].

Microorganisms employ different mechanisms to tolerate and detoxify paraquat, including the induction of antioxidant enzymes such as superoxide dismutase (SOD), which helps mitigate the oxidative stress caused by the herbicide [49]. Additionally, some bacterial species have been reported to metabolically transform paraquat through biodegradation pathways, in some cases using it as a source of carbon or nitrogen during microbial metabolism [29,92,93]. These findings highlight the potential of indigenous soil microorganisms as promising agents for the bioremediation of paraquat-contaminated environments.

The microbial biodegradation of the herbicide paraquat is complex and slow due to its high environmental persistence, mainly in soils, the great stability of its bipyridylic chemical structure, and its high toxicity, mediated by the generation of reactive oxygen species [31,94]. However, bacterial strains capable of degrading or removing it from the environment have been reported [46].

Table 2 shows recent research on paraquat degradation/removal by different bacterial strains. As shown in the table, the removal of the herbicide paraquat exhibits considerable variability, which depends mainly on the strain studied, the initial concentration of the herbicide, and the duration of the assay. Therefore, for comparative purposes, the paraquat removal rate was calculated for each study in this work. In this study, *C. zhejiangensis* CEIB S4-3 showed a removal percentage of 40.83% in 48 h at a concentration of 12 mg/L, and a removal rate of 0.10 mg/L·h.

This paraquat elimination rate is lower than that calculated for *Achromobacter* sp. strain BUK-BCH-TQ1, which was 2.09 mg/L·h after 120 h at a concentration of 276 mg/L. Likewise, compared to the *Bacillus subtilis* TISTR 1248 and *Pseudomonas putida* TISTR 1522 strains, which achieved paraquat elimination percentages greater than 98% at 10 mg/L in less than 10 h, demonstrating faster elimination, with an elimination rate of 0.99 mg/L·h.

On the other hand, the performance of *C. zhejiangensis* CEIB S4-3 in the elimination of paraquat is comparable to or even superior to that of other reported bacterial strains, such as *Bacillus subtilis*, *Pseudomonas geniculata* PQ01, *Pseudomonas putida*, *Aeromonas veronii* NK67 and the consortium, composed of seven bacterial strains, *Sphingomicrobium marinum*, *Ferrovibrio xuzhouensis*, *Azospirillum lipoferum*, *Altererythrobacter xinjiangensis*, *Xanthobacter autotrophicus*, and *Azospirillum amazonense*, where the degradation rates were 0.016, 0.06, 0.016, 0.07 and 0.01 mg/L·h, respectively, because in these studies the strains require longer times (144–840 h) for the paraquat elimination.

Based on the degradation rate, the *C. zhejiangensis* CEIB S4-3 strain has the potential to remove paraquat in vitro, but its degradation efficiency requires improvement. Recent studies have successfully increased paraquat removal through strategies such as the addition of co-substrates (sucrose) and electron-accepting compounds like anthraquinone-2,6-disulfonate (AQDS), increasing paraquat removal from 18.6% with AQDS supplementation alone to 100% after 5 days with sucrose and AQDS supplementation [95]. Furthermore, the immobilization of microorganisms on supports has been reported as an effective alternative to increase pesticide removal in bacterial models [96]. Immobilization of *Pseudomonas putida* on coconut fiber biochar [97] and corn cob biochar [6], as well as on nanoceramic supports [45], has increased paraquat removal rates to over 90%. Future studies could evaluate these strategies to enhance the paraquat removal efficiency of the *C. zhejiangensis* CEIB S4-3 strain.

**Table 2:** Recent investigations on paraquat elimination by bacterial species.

Bacterial Strain	Concentration (mg/L)	Time (h)	Elimination Percentage (%)	Elimination Rate (mg/L·h)	Reference
*Achromobacter* sp. strain BUK-BCH-TQ1	276	120	91.01	2.09	[92]
*Pseudomonas putida* TISTR 1522	10	24	4	0.016	[45]
*Bacillus subtilis* TISTR 1248					
*Pseudomonas putida*	69.76	72	47.29	0.46	[98]
Unidentified (PQ-1, PQ-2, and PQ-3)	50	120	100	0.42	[99]
*Pseudomonas putida*	25	24	21	0.22	[6]
*Micrococcus* sp. S2	40	48	20	0.17	[100]
*Bacillus aryabhattai* MoB09	10	72	100	0.14	[101]
*Caballeronia zhejiangensis* CEIB S4-3	12	48	40.83	0.10	This work
*Aeromonas veronii* NK67	20	144	53.4	0.07	[102]
*Pseudomonas geniculata* PQ01	50	288	33.4	0.06	[95]
*Pseudomonas putida*	30	48	6.7	0.042	[97]
*Sphingomicrobium marinum*, *Ferrovibrio xuzhouensis*, *Azospirillum lipoferum*, *Altererythrobacter xinjiangensis*, *Xanthobacter autotrophicus*, and *Azospirillum amazonense*	10	840	95.69	0.01	[91]

Based on the previous information, it has been demonstrated that several bacterial genera have the ability to develop mechanisms of resistance and degradation of paraquat, making them promising candidates for bioremediation strategies in contaminated environments.

In this study, genomic analysis allowed the identification of genetic sequences involved in different resistance mechanisms and bacterial biodegradation of the paraquat herbicide. Paraquat is a well-known pollutant that induces severe oxidative stress in bacteria. Exposure to paraquat generates various adverse effects in bacteria, among the most important being the generation of reactive oxygen species, mainly the superoxide radical (O_2_^−^) and hydrogen peroxide (H_2_O_2_), inducing an oxidative stress condition in bacterial cells. This condition unchain damage to cellular membranes, proteins and DNA [103,104], this damage compromising cellular viability, metabolism, and triggering stress responses in bacteria. In agreement with this, the genome of *C. zhejiangensis* CEIB S4-3 contains genes encoding antioxidant enzymes such as superoxide dismutase, catalase, flavodoxins, and peroxiredoxins, which are associated with ROS detoxification [48,105,106].

Additionally, transcriptional regulators such as SoxR, SoxS, OxyR, and OxyS were identified, which are key components of the global response to oxidative stress [57,59,60,105,107,108,109]. These regulators coordinate detoxification, repair processes, and stress adaptation. For example, SoxR and OxyR have been reported to participate in the response to paraquat in *Paraburkholderia xenovorans* LB400 [60], while in *Stenotrophomonas maltophilia*, SoxR regulates efflux systems such as MfsA, influencing paraquat susceptibility [58].

Consistent with these mechanisms, genes encoding efflux systems of the MFS and RND families were also identified, which are associated with the detoxification of toxic compounds, including paraquat [75,110]. These systems may reduce intracellular accumulation of the herbicide and contribute to bacterial resistance.

Furthermore, genes encoding the paraquat-inducible system (PqiABC) were detected. This system has been reported to play an important role in maintaining membrane integrity under oxidative stress conditions [78,79,80,111,112]. The PqiABC complex forms a transmembrane channel that connects the inner and outer membranes, facilitating phospholipid transport and contributing to membrane stability during paraquat-induced stress.

Regarding biodegradation, genomic analysis revealed the presence of genes encoding enzymes potentially involved in paraquat transformation, including cytochrome P450, flavin-containing monooxygenases, and dioxygenases [46,88,113,114,115,116]. These enzymes are known to catalyze oxidation, demethylation, and ring-cleavage reactions in aromatic compounds. Cytochrome P450 enzymes have been associated with N-demethylation processes, while monooxygenases may initiate oxidative modifications of the bipyridyl structure [88,113]. Dioxygenases, in turn, can catalyze the incorporation of oxygen into aromatic rings, facilitating their cleavage and subsequent mineralization [114,115,116,117]. Given that paraquat contains aromatic pyridine rings, these enzymes may contribute to its degradation [118,119].

The results of this study indicate that the resistance and degradation capacity of *C. zhejiangensis* CEIB S4-3 are supported by a combination of mechanisms, including antioxidant defense, transcriptional regulation, efflux systems, membrane repair, and enzymatic transformation. These findings suggest that paraquat biodegradation is not a single-enzyme-dependent process but rather a coordinated complex [46,113], supporting the potential application of this strain in bioremediation strategies. However, it is important to validate the expression of these genes in the presence of the herbicide paraquat; accordingly, in subsequent studies, the evaluation of their expression levels under herbicide exposure is proposed through a transcriptomic study.

## 5. Conclusions

The results of this study demonstrate that the bacterial strain *C. zhejiangensis* CEIB S4-3 is capable of resisting both the commercial formulation Gramoxone^®^ and analytical-grade paraquat. In TSA, the strain exhibited growth in the presence of concentrations of up to 400 mg/L of Gramoxone^®^ and 800 mg/L of analytical-grade paraquat. In TSB, a bacteriostatic effect was observed during the first 36 h, followed by an exponential growth phase that extended up to 60 h. In minimal medium (MSM), the strain was able to survive in the presence of 1, 3, 5, 10, and 15 mg/L of Gramoxone^®^ or analytical-grade paraquat. However, at 5, 10, and 15 mg/L of Gramoxone^®^, a significant decrease in population density was observed, whereas in the case of analytical-grade paraquat, growth inhibition occurred only at 10 and 15 mg/L. Additionally, the strain was able to remove 40.83% of paraquat at an initial concentration of 12 mg/L after 48 h of incubation. Genomic analysis revealed the presence of genes encoding enzymes potentially involved in the transformation of xenobiotic compounds, such as cytochrome P450, monooxygenases, dioxygenases, and paraquat-inducible proteins, suggesting their possible involvement in paraquat degradation mechanisms. These findings highlight the potential of *C. zhejiangensis* CEIB S4-3 to resist and potentially degrade paraquat, contributing to the understanding of the molecular mechanisms underlying paraquat resistance and bacterial degradation. Given that *C. zhejiangensis* CEIB S4-3 has been previously reported by our research group to possess capacity to degrade various pesticides, this strain represents a promising candidate to metabolize paraquat, highlighting its potential application in bioremediation strategies for agricultural soils contaminated by this herbicide.

## Figures and Tables

**Table 1:** Paraquat resistance sequences in the *C. zhejiangensis* CEIB S4-3 genome.

Process	Name	Gen	Description	Genome Location	Clusters of Orthologous Genes (COGs)	Functional Annotation (KEEG)	NCBI-BLAST Results			
							Query Cover (%)	E Value	Identity (%)	Orthologous Proteins NCBI Accession
Sensing and activation of the oxidative stress contention	Oxidative stress regulatory proteins [56,57,58,59,60,61]	*sox*R	SoxR is a transcriptional regulator in bacteria that belongs to the MerR-type transcriptional regulator family. It is part of the SoxRS regulon, serving as a sensor for reactive oxygen species such as superoxide radicals (O_2_^−^) and activating the expression of the soxS gene.	Contig 2 JSBM01000073.1:422998-423417	COG0789: [K] Transcription-DNA-binding transcriptional regulator, MerR family [Transcription]	K13639: MerR family transcriptional regulator, redox-sensitive transcriptional activator SoxR. K19591: MerR family transcriptional regulator, copper efflux regulator.	100	1 × 10^−74^	92.14	MerR family transcriptional regulator [*Caballeronia* sp. LZ034LL] WP_309806998.1
				Contig 21 JSBM01000092.1:43458-43817	COG0789: [K] Transcription-DNA-binding transcriptional regulator, MerR family [Transcription]	K13639: MerR family transcriptional regulator, redox-sensitive transcriptional activator SoxR. K19591: MerR family transcriptional regulator, copper efflux regulator.	100	1 × 10^−82^	100	MULTISPECIES: Cu(I)-responsive transcriptional regulator [*Burkholderiaceae*] WP_029672610.1
		*sox*S	SoxS is a transcriptional regulator in bacteria that belongs to the AraC-type transcriptional regulator family. It is part of the SoxRS regulon, serving as an activator of detoxification genes in the presence of reactive oxygen species such as superoxide radicals (O_2_^−^). In paraquat exposure, SoxS activates antioxidant defense through the expression of antioxidant enzymes, such as superoxide dismutase, efflux systems, and oxidative damage repair.	Contig 33 JSBM01000076.1:561-1496	COG2207: [K] Transcription-AraC-type DNA-binding domain and AraC-containing proteins	K18991: mtrA AraC family transcriptional regulator, activator of mtrCDE	100	0.0	99.68	MULTISPECIES: AraC family transcriptional regulator [*Burkholderiaceae*] WP_075583584.1
	Oxidative stress regulatory proteins [59,60,61]	*oxy*R	OxyR is a transcriptional regulator in bacteria that belongs to the LysR-type transcriptional regulator family. OxyR serves as a sensor, activator and regulator in response to reactive oxygen stress caused by H_2_O_2_. OxyR induces the expression of catalases and peroxidases, as well as the expression of the OxyS transcriptional regulator in bacteria.	Contig 23 JSBM01000134.1:3637-4530	COG0583: [K] Transcription-DNA-binding transcriptional regulator, LysR family	KO: no assigned	100	1 *×* 10^−163^	91.95	LysR family transcriptional regulator [*Caballeronia calidae*] WP_062610805.1
		*oxy*S	OxyS is a small transcriptional regulator in bacteria that belongs to the LysR-type transcriptional regulator family. OxyS together with OxyR coordinates oxidative stress response in bacteria, including H_2_O_2_ degradation and efflux.	Contig 53: JSBM01000035.1:2621-3337	COG0583: [K] Transcription-DNA-binding transcriptional regulator, LysR family	K19338: nac LysR family transcriptional regulator, nitrogen assimilation regulatory protein	100	3 × 10^−169^	100	MULTISPECIES: LysR family transcriptional regulator [*Caballeronia*] WP_008345181.1
Antioxidant enzymes	Superoxide dismutases	*sod*B	Iron Superoxide dismutase (FeSOD) is a cytosolic enzyme member of a larger class of superoxide dismutase (SOD) proteins. SODs protect cells from reactive oxygen species (ROS), converting the harmful molecule superoxide (O_2_^−^) into less toxic substances, such as H_2_O_2_ [49,62,63].	Conting 12: JSBM01000154.1:66531-67103	COG0605: [P] Inorganic ion transport and metabolism-Superoxide dismutase	K04564: SOD2 superoxide dismutase, Fe-Mn family [EC:1.15.1.1]	100	2 × 10^−129^	91.	MULTISPECIES: superoxide dismutase [Fe] [*Burkholderiaceae]* WP_087658105.1
	Catalases	*cat*	Catalases are antioxidant enzymes widely distributed in aerobic organisms. Its main function is to decompose hydrogen peroxide (H_2_O_2_)—a potentially toxic reactive species of oxygen— into water (H_2_O) and molecular oxygen (O_2_), thus protecting cells from oxidative damage [62,63,64,65].	Contig 2: JSBM01000073.1:166778-167944	COG0753: [P] Inorganic ion transport and metabolism-Catalase	K03781: katE, CAT, catB, srpA; catalase [EC:1.11.1.6]	100	0.0	93.47	Catalase [*Caballeronia pedi*] WP_061172810.1
				Contig 4: JSBM01000097.1:147207-148106	COG0753: [P] Inorganic ion transport and metabolism-Catalase	K03781: katE, CAT, catB, srpA; catalase [EC:1.11.1.6]	100	0.0	95.67	Catalase family peroxidase [*Caballeronia jiangsuensis*] WP_268807886.1
				Contig 79: JSBM01000019.1:9073-10236	COG0753: [P] Inorganic ion transport and metabolism-Catalase	K03781: katE, CAT, catB, srpA; catalase [EC:1.11.1.6]	100	0.0	98.71	Catalase HPII [*Caballeronia catudaia*] WP_268807886.1
	Flavodoxins	*fld*	Flavodoxin participates in multiple redox and metabolic processes in bacteria, maintaining cellular redox balance. Expression of cyanobacterial flavodoxin in several bacteria increased tolerance to paraquat and other ROS-generating herbicides [48,66].	Contig 43: JSBM01000098.1:15425-15730	COG0655: [C] Energy production and conversion-Multimeric flavodoxin WrbA	K03809: wrbA NAD(P)H dehydrogenase (quinone) [EC:1.6.5.2]	88	6 × 10^−58^	100	MULTISPECIES: flavodoxin family protein [*Caballeronia*] WP_175979594.1
	Peroxiredoxins [67,68]	*ahp*E	Peroxiredoxins are peroxidase enzymes with a conserved cysteine residue in the active site (thiol-dependent antioxidant enzymes), that catalyze the reduction of hydrogen peroxide (H_2_O_2_) and other organic hydroperoxides [69].	Contig 72: JSBM01000026.1:7875-8525	COG0450: [V] Defense mechanisms-Alkyl hydroperoxide reductase subunit AhpC (peroxiredoxin)	K24158: prx thioredoxin-dependent peroxiredoxin [EC:1.11.1.24]	100	2 × 10^−154^	95.85	MULTISPECIES: peroxiredoxin [Unclassified *Caballeronia*] WP_309838052.1
				Contig 60: JSBM01000105.1:5719-6165	COG0450: [V] Defense mechanisms-Alkyl hydroperoxide reductase subunit AhpC (peroxiredoxin)	K24126: ahpC lipoyl-dependent peroxiredoxin subunit C [EC:1.11.1.28]	100	4 × 10^−100^	93.96	MULTISPECIES: peroxiredoxin [*Paraburkholderia*] WP_007182021.1
		*ahp*C	Peroxiredoxins like proteins with two cysteine residues in the active site, catalyze the reduction of hydrogen peroxide (H_2_O_2_) and other organic hydroperoxides [70].	Contig 8: JSBM01000115.1:202434-202898	COG0450: [V] Defense mechanisms-Alkyl hydroperoxide reductase subunit AhpC (peroxiredoxin)	K24119: ahpC NADH-dependent peroxiredoxin subunit C [EC:1.11.1.26]	100	5 × 10^−105^	92.9	Alkyl hydroperoxide reductase subunit C [*Caballeronia concitans*] WP_040051042.1
Efflux systems	Major Facilitator Superfamily efflux pumps	*mfs*	Major facilitator superfamily (MFS) is a group of efflux system implicated in antibiotics and xenobiotics resistance. This membrane proteins are used by bacteria to export toxic substances out of the cell [71,72].	Contig 3: JSBM01000003.1:406758-408218	COG0477: MFS family permease	K08137: galP MFS transporter, SP family, galactose: H+ symporter	100	0.0	94.32	Sugar porter family MFS transporter [*Paraburkholderia hospita*] WP_341352358.1
				Contig 9: JSBM01000084.1:175652-176593	COG0477: MFS family permease	K07552: bcr, tcaB MFS transporter, DHA1 family, multidrug resistance protein	100	6 × 10^−166^	91.4	Bcr/CflA family multidrug efflux MFS transporter [*Caballeronia* sp. RCC_10] WP_370885160.1
				Contig 18: JSBM01000085.1:73476-74246	COG0477: MFS family permease	K03446: MFS transporter, DHA2 family, multidrug resistance protein K07786: MFS transporter, DHA2 family, multidrug resistance protein	93	7 × 10^−119^	91.63	DHA2 family efflux MFS transporter permease subunit [*Caballeronia* sp. NK8] BCQ25768.1
				Contig 43: JSBM01000098.1:21627-22805	COG0477: MFS family permease	K08166: MFS transporter, DHA2 family, methylenomycin A resistance protein	100	0.0	97.19	MFS transporter [*Caballeronia temeraria*] WP_061164780.1
				Contig 68: JSBM01000048.1:4259-5044	COG0477: MFS family permease	K08166: MFS transporter, DHA2 family, methylenomycin A resistance protein	100	1 × 10^−125^	100	DHA2 family efflux MFS transporter permease subunit [*Caballeronia zhejiangensis*] WP_033536325.1
	Resistance–Nodulation–Division (RND) family transporters	*acr*	RND family pumps are a major group of multidrug efflux systems in bacteria, RND transporters actively expel antibiotics, toxins, and detergents out of bacterial cells [73,74,75,76].	Contig 1: JSBM01000045.1:509896-513048	COG0841: [V] Defense mechanisms-Multidrug efflux pump subunit AcrB	K18138: acrB, mexB, adeJ, smeE, mtrD, cmeB multidrug efflux pump	99	0.0	95.59	Efflux RND transporter permease subunit [*Caballeronia glebae*] WP_086969125.1
				Contig 2: JSBM01000073.1:30618-33743	COG0841: [V] Defense mechanisms-Multidrug efflux pump subunit AcrB	K18138: acrB, mexB, adeJ, smeE, mtrD, cmeB multidrug efflux pump	100	0.0	95.2	MULTISPECIES: efflux RND transporter permease subunit [*Caballeronia*] WP_309883707.1
				Conting 3: JSBM01000003.1:54466-57597	COG0841: [V] Defense mechanisms-Multidrug efflux pump subunit AcrB	K18138: acrB, mexB, adeJ, smeE, mtrD, cmeB multidrug efflux pump	100	0.0	93.42	Efflux RND transporter permease subunit [*Burkholderia* sp. 8Y] WP_159839562.1
				Conting 8: JSBM01000115.1:131291-134413	COG0841: [V] Defense mechanisms-Multidrug efflux pump subunit AcrB	K18902: bpeF multidrug efflux pump	100	0.0	92.8	Efflux RND transporter permease subunit [*Caballeronia udeis*] WP_062088636.1
				Conting 30: JSBM01000096.1:75200-77524	COG0841: [V] Defense mechanisms-Multidrug efflux pump subunit AcrB	K18902: bpeF multidrug efflux pump	100	0.0	96.52	Efflux RND transporter permease subunit [*Burkholderia* sp. THE68] WP_162065286.1
Membrane remodeling/oxidative damage repair	Paraquat inducible proteins	*pqi*A	Cell membrane lipid transport in response to oxidative stress induced by agents such as paraquat [77,78,79,80].	Contig 2: JSBM01000073.1:181562-182137	COG2995: [S] Function unknown Uncharacterized paraquat-inducible protein A	KO: no assigned	100	4 × 10^−121^	99.48	MULTISPECIES: paraquat-inducible protein A [*Caballeronia*] WP_175979801.1
		*pqi*B		Contig 1: JSBM01000073.1:182870-184546	COG3008: [S] Function unknown Uncharacterized paraquat-inducible protein A	KO: no assigned	100	0.0	93.56	PqiB family protein [*Caballeronia* sp. NK8] WP_213226993.1
		*pqi*C		Contig 1: JSBM01000045.1:517469-518050	COG: No assigned	K09857: intermembrane transport lipoprotein PqiC	100	3 × 10^−92^	100	MULTISPECIES: PqiC family protein [*Caballeronia*] WP_008348482.1
Paraquat degradation enzymes	Cytochrome P450	*cyp*	Cytochrome P450 is a protein that has an oxidoreductase activity and participates in oxidation, hydroxylation, demethylation, or reduction of xenobiotics. It has been proposed that CytP450 is involved in the N-demethylation of paraquat [46,81,82,83,84].	Contig 78: JSBM01000057.1:2829-5099	COG2124: [V] Defense mechanisms-Cytochrome P450	K00490: CYP4F; cytochrome P450 family 4 subfamily F [EC:1.14.14.1]	100	0.0	93	MULTISPECIES: cytochrome P450/oxidoreductase [Unclassified *Caballeronia]* WP_061116131.1
	Monooxygenases	*fmo*	Flavin-monooxygenases catalyzes the incorporation of an oxygen atom into xenobiotics molecules using NADH or NADPH as electron donors. In paraquat degradation monooxygenases could have a role in the N-demethylation, hydroxylation or the initial oxidative modification of the bipyridyl structure, before ring cleavage reactions [85,86,87,88].	Contig 6: JSBM01000032.1:254321-254656	COG2072: [P] Inorganic ion transport and metabolism-Predicted flavoprotein CzcO associated with the cation diffusion facilitator CzcD	K22879: FAD-dependent urate hydroxylase [EC:1.14.13.113]	100	5 × 10^−57^	96.43	Flavin-containing monooxygenase [*Caballeronia temeraria*] WP_061161686.1
	Dioxygenases		Dioxygenases catalyze the incorporation of two atoms of molecular oxygen into aromatic and heterocyclic xenobiotics. In paraquat degradation, dioxygenases could open the aromatic ring and cleave the molecule into smaller organic acids [46,85,87].	Contig 4: JSBM01000097.1:104935-105897	COG2514: [Q] Secondary metabolites biosynthesis, transport and catabolism-Catechol-2,3-dioxygenase	K00446: catechol 2,3-dioxygenase [EC:1.13.11.2]	100	0	95.95	VOC family protein [*Caballeronia cordobensis*] WP_045456136.1
		*catA*		Contig 22: JSBM01000058.1:62049-62981	COG3485: [Q] Secondary metabolites biosynthesis, transport and catabolism-Protocatechuate 3,4-dioxygenase beta subunit	K03381: catechol 1,2-dioxygenase [EC:1.13.11.1]	100	0.0	92.6	Catechol 1,2-dioxygenase [*Caballeronia calidae*] WP_062610444.1
		*doda*		Contig 40: JSBM01000041.1:17526-18305	COG3384: [Q] Secondary metabolites biosynthesis, transport and catabolism-Aromatic ring-opening dioxygenase, catalytic subunit, LigB family	K15777: 4,5-DOPA dioxygenase extradiol [EC:1.13.11.-]	100	7 × 10^−126^	96.35	MULTISPECIES: DODA-type extradiol aromatic ring-opening family dioxygenase [*Caballeronia*] WP_034473915.1
	Peroxidases	*dyp*	Dye-decolorizing peroxidases. Heme-dependent peroxidases that catalyze oxidation reactions using hydrogen peroxide (H_2_O_2_) as an oxidant. DyP-type peroxidases have also been reported to possess activities in addition to peroxidase function, including hydrolase or oxidase activity [89,90].	Contig 6: JSBM01000032.1:136224-136841	COG2837: [P] Inorganic ion transport and metabolism-Periplasmic deferrochelatase/peroxidase EfeB	K07223: porphyrinogen peroxidase [EC:1.11.1.-]|(RefSeq) putative dyp-type peroxidase family protein	100	3 × 10^−144^	95.15	Dyp-type peroxidase [*Caballeronia novacaledonica*] WP_238292368.1

## Data Availability

The original contributions presented in this research are included in the article. Further inquiries can be directed to the corresponding authors.

## Supplementary Materials

The following supporting information can be downloaded at: https://www.mdpi.com/article/10.3390/toxics14050405/s1, Figure S1: Paraquat UHPLC calibration curve; Figure S2: Chromatograms of paraquat in the control experiments (paraquat + MSM); Figure S3: Chromatograms of paraquat degradation kinetics in presence of the bacterial strain *C. zhejiangensis* CEIB S4-3.
