# Supplementary material for: Assessment of Paraquat Resistance and Degradation Potential in Caballeronia zhejiangensis CEIB S4-3: The Genomic Analysis Reveals Hints About Resistance and Degradation Mechanisms
Source: Toxics. 2026 May 8;14(5):405. doi: 10.3390/toxics14050405 (PMC13210998; doi:10.3390/toxics14050405)
Supplement: Supplementary file 1 [file toxics-14-00405-s001.zip › toxics-4238012-supplementary.pdf]

Supplementary material

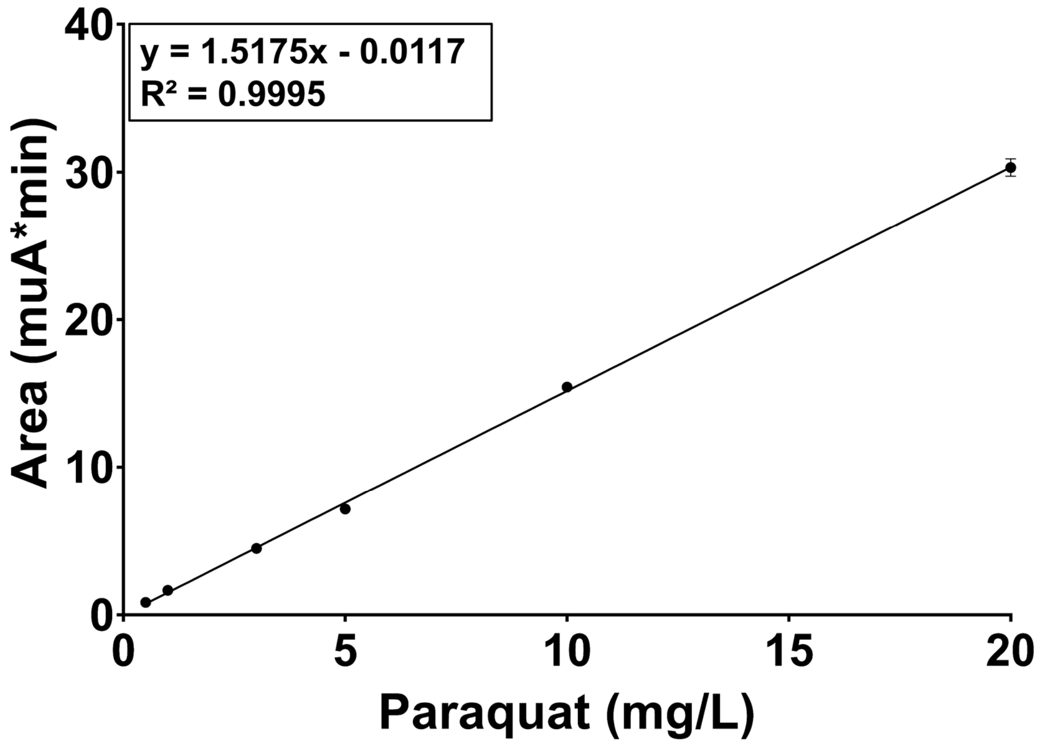

Figure S1. Paraquat UHPLC calibration curve. Paraquat concentration range 0.5 to 20 mg/L.

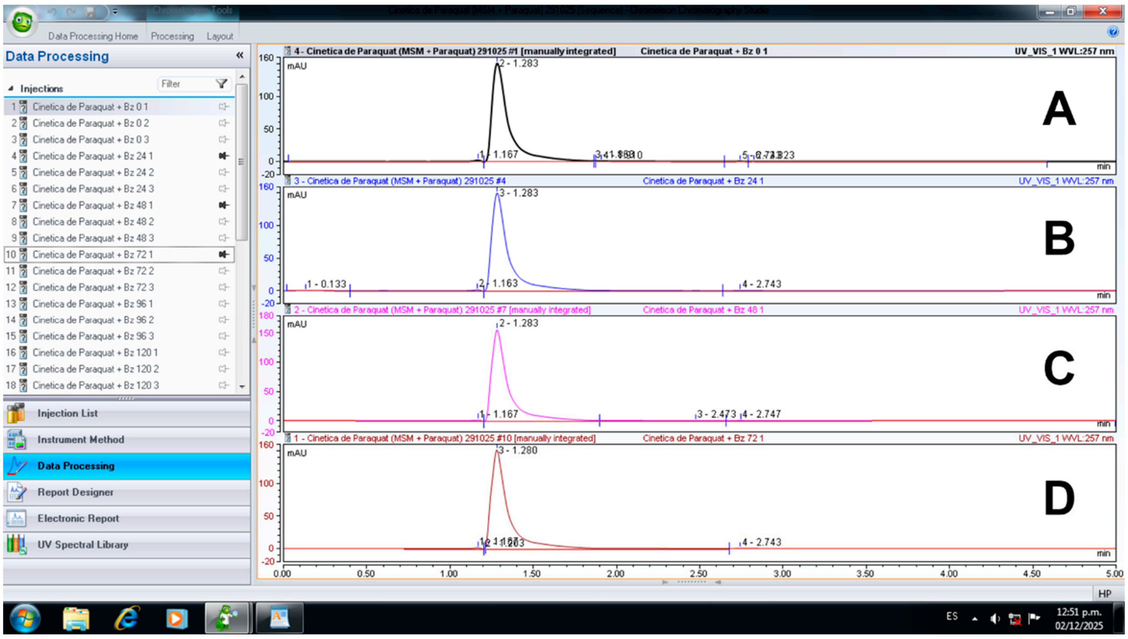

Figure S2. Cromatograms of paraquat in the control experiments (paraquat + MSM). A) Starting time (0 h), B) 24 h of incubation, C) 48 h of incubation and D) 72 h of incubation.

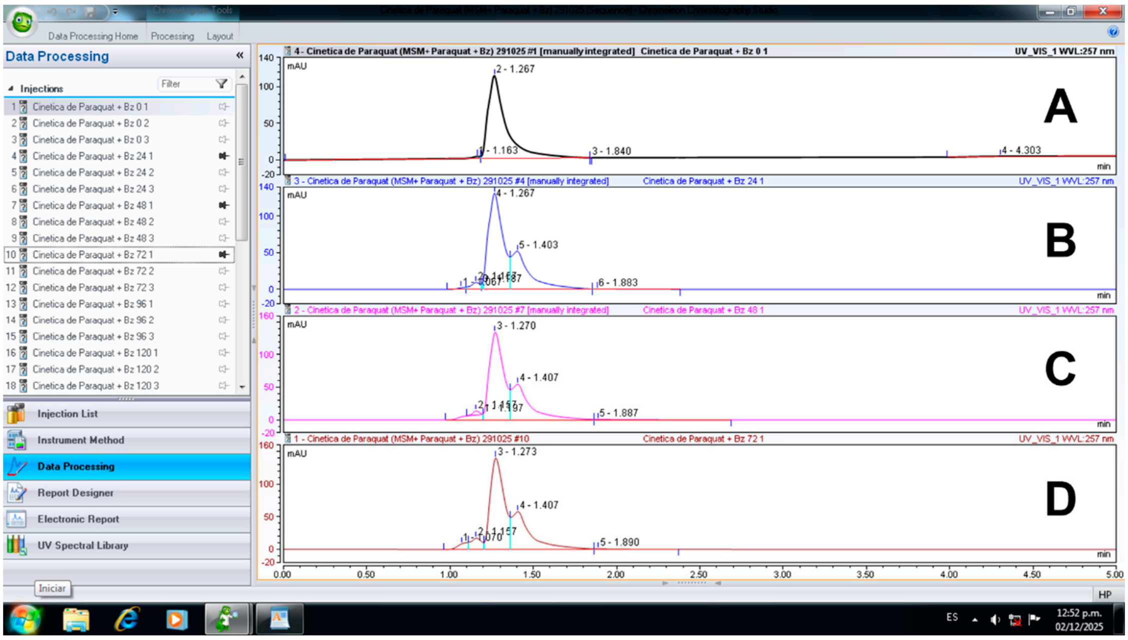

Figure S3. Cromatograms of paraquat degradation kinetics in presence of the bacterial strain *C. zhejiangensis* CEIB S4-3. A) Starting time (0 h), B) 24 h of incubation, C) 48 h of incubation and D) 72 h of incubation.
